# Three‐dimensional digitization of the arid land plant *Haloxylon ammodendron* using a consumer‐grade camera

**DOI:** 10.1002/ece3.4126

**Published:** 2018-05-02

**Authors:** Hongyu Huang, Hao Zhang, Chongcheng Chen, Liyu Tang

**Affiliations:** ^1^ Key Laboratory of Spatial Data Mining & Information Sharing of Ministry of Education Fuzhou University Fuzhou China; ^2^ National Engineering Research Centre of Geospatial Information Technology Fuzhou University Fuzhou China

**Keywords:** 3D modeling, computer vision, desert plant, individual plant, LIDAR, photogrammetry, point cloud, terrestrial laser scanner

## Abstract

Plant structural parameters are important for ecological studies and for monitoring the environment. Terrestrial laser scanning has become a widely accepted technique for acquiring accurate high‐density three‐dimensional information about plant surfaces; however, this instrument is expensive, technically challenging to operate, heavy, and difficult to transport to hard‐to‐reach areas such as dense forests and undeveloped areas without easy vehicle access. Using *Haloxylon ammodendron*, a plant widely distributed in arid lands, as an example, we used a consumer‐grade handheld camera to take a series of overlapping images of this plant. Computer vision and photogrammetric software were used to reconstruct highly detailed three‐dimensional data of the plant surface. This surface data was compared to the point cloud of the plant acquired from concomitant terrestrial laser scanning. We demonstrated that the accuracy and degree of completeness of the image‐derived point clouds are comparable to that of laser scanning. Plant structural parameters (such as tree height and crown width) and three‐dimensional models extracted from the point clouds also agree well with a relative difference of less than 5%. Our case study shows that a common camera and image processing software can be an affordable, highly portable, and viable option for acquiring accurate and detailed high‐density and high‐resolution three‐dimensional information about plant structure in the environment. This digitization technique can record the plant and its surrounding environment effectively and efficiently, and it can be applied to many ecological fields and studies.

## 1 INTRODUCTION

Plant structural parameters are key metrics for ecological research and applications. Obtaining accurate plant structural parameters (such as leaf area, stem diameter, stem form and taper, plant height, and crown width) is important for a wide variety of applications within the fields of forestry and ecology and for environmental studies (Dandois and Ellis, 2013). Traditional manual measurements are both labor‐intensive and time‐consuming; in the past decade, terrestrial laser scanning (TLS, also called ground‐based or tripod‐based laser scanning) has been gradually accepted as an efficient noncontact method for tree measurements and inventorying forests at both individual tree and plot‐level scales (Liang et al., 2016; Newnham et al., 2015; Wallace, Hillman, Reinke, & Hally, 2017). Point cloud data acquired from TLS provide accurate and detailed information about plant size and architecture. However, TLS has some disadvantages and limitations: It is relatively expensive and difficult to carry around and requires training to set up and operate properly.

Recent advancements in computer vision and photogrammetry have made consumer‐grade cameras an affordable alternative for capturing reality. Detailed three‐dimensional (3D) surface information of the scene can be reconstructed from overlapping images taken by the camera from different viewpoints. Some examples of recent applications include single‐tree and plot‐level 3D modeling (Bauwens et al., 2017; Liang et al., 2014; Mikita, Janata, & Surový, 2016; Miller, Morgenroth, & Gomez, 2015) and topographic geomorphlogical mapping (Micheletti, Chandler, & Lane, 2015; Smith, Carrivick, & Quincey, 2016).

Saxaul (*Haloxylon ammodendron*) is a drought‐resistant plant species distributed widely in the arid lands of the Middle East and Central Asia. Its size ranges from a large shrub to a small tree (Wikipedia, 2017). As the dominant species in some desert ecotones, its sap flow, water consumption, and transpiration have been extensively studied, and its ecological functions are closely related to its physiological characteristics (Cao, Chen, Wang, Wang, & Wang, 2013; Sun, Zhou, Li, & Li, 2010). Therefore, the ability to quickly and precisely measure the 3D structural parameters of this and other desert species would be beneficial. Although laser scanning is widely used in forest and ecological applications, to the best of our knowledge, there are very few reported studies that document desert plants in 3D using either TLS or computer vision techniques.

Due to its high accuracy and fine resolution, laser scanning has been used in many studies to produce high‐quality ground truth data. Most recently, in a surface vegetation biomass study, Wallace et al. (2017) compared 3D point clouds derived from digital images to 3D point clouds collected through TLS surveys; Knapitsch, Park, Zhou, and Koltun (2017) created a benchmark for image‐based 3D reconstruction in which video sequences were used as input; the resulting point cloud was compared to the point cloud produced from a Faro scanner. Schöps et al. (2017) also used a high‐precision laser scanner to record the ground truth scene geometry for their benchmark. We took a similar approach in this study, using 3D data obtained from TLS as a reference to evaluate the data derived from the photogrammetric software.

The main goal of this study is to determine whether a computer vision‐based technique can be used to obtain accurate high‐resolution 3D information from saxaul (*Haloxylon ammodendron*). Specifically, we would like to answer the following questions. (1) Can 3D surface points be obtained using computer vision and photogrammetry to process overlapping photographs taken from around the plant with an off‐the‐shelf consumer‐grade camera? (2) How does this image‐derived point cloud data compare to point clouds derived from the established laser scanning technique in terms of accuracy and completeness? (3) How does the plant structural information derived from the point clouds produced using the two techniques compare?

The rest of the study is organized as follows: The study site, data acquisition methods, and processing steps are described in Section 2; data comparisons are shown in Section 3, and we present a discussion in Section 4 and a summary in Section 5.

## 2 STUDY AREA AND DATA ACQUISITION

### 2.1 Study area

The experiment was conducted in BeiShaWo Experimental Field (44^o^25′56.5″N and 87^o^54′13″E with an elevation of 468 m above sea level) at the Fukang Desert Ecological Research Station (Cao et al., 2013); the study site was located in the southern part of the Gurbantünggüt Desert, the second largest desert in China. *Haloxylon ammodendron* is the dominant species in the region and is widely distributed. Surveys of the area's vegetation showed that the plant density was approximately 600 plants/hm^2^; the average plant height was 1.8 to 1.92 m, and the average crown size was 2 m by 1.75 m (Cao et al., 2013; Sun et al., 2010).

### 2.2 TLS data acquisition and processing

The study site was first scanned with a Riegl VZ‐400 (Riegl Laser Measurement Systems GmbH, Horn, Austria) laser scanner on 8 August 2015 to obtain detailed information about plants in the plot. The transmitted laser pulse is in the near‐infrared spectrum (with a wavelength of 1,550 nm), and the scanner is accurate to 3 mm at a 100 m distance. The normal working measurement range is dependent on the target reflectivity; for example, at 90% reflectivity, the range can be up to 600 m; with 20% reflectivity, it is 280 m. The scanner weighs approximately 10 kg (excluding the battery).

**Figure 1:**
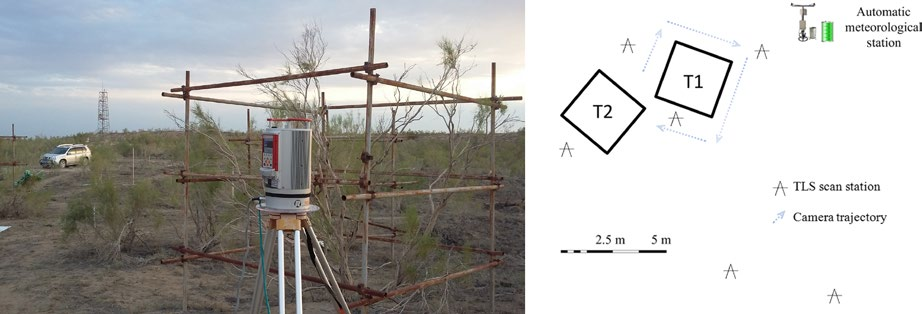
The study site (left: photograph showing TLS being used at the site; right: schematic drawing showing the positions of the plants and laser scans, as well as the camera trajectories; T1 represents tree 1)

There are seven saxaul plants growing in this plot that are being continuously monitored for stem sap flow along with other long‐term ecological observations. Each individual plant is protected within a cage‐like structure made of steel pipes (Figure 1). The study area was laser‐scanned from 10 different positions to get comprehensive information about the site; Figure 1 shows six scan positions surrounding trees 1 and 2. A full‐view (360 degrees by 100 degrees) scan was performed at each scan station, and the angular resolution was set to 0.04 degrees. At a 10 m distance to the scanner, this 0.04‐degree angular resolution corresponds to a point interval of 7 mm. Reflective disks with a 5 cm diameter were set up in the scene before scanning, and care was taken to make sure that these disks could be seen from at least two scan positions.

The weather was cloudy with some wind. For each full‐view laser scan at 0.04 degrees, more than 10 million data points were acquired. Scan time in the field was around 10 min per station (a panoramic scan could be completed in less than 4 min, the reflective disk targets were located, and performing a fine scan took approximately 5 min).

Reflective disks being scanned were identified in the resulting point cloud and used as tie points for scan‐to‐scan registration; registration results of better than 1 cm were achieved for all of the scans. After registration, all of the scan data were put into the same project coordinate system, and data from all scan positions were combined to get full coverage of the study objects. The data were registered and combined using the RiscanPRO software that was supplied with the laser scanner. Furthermore, outliers and noise were also removed using the software tool. Basic data processing was usually finished in half an hour. The TLS point cloud served as a reference for evaluating the image‐based point cloud.

### 2.3 Image acquisition and processing

Image data were acquired the day after laser scanning. The sky was overcast when photographs were taken. A Nikon D600 digital camera and AF‐S Nikkor 24‐85 mm f/3.5‐4.5G ED VR lens (Nikon Corp., Tokyo, Japan) were used. The total weight (the camera body, battery, and lens) was 1.2 kg.

A series of images were acquired of tree 1. The operator took a picture of the plant from a specific position and then moved to the next position by a small step. Care was taken to make sure that there were many overlaps (at least 60%) between two consecutive photographs. The camera was held by an operator at approximately eye level (170 cm above ground) and pointed toward the tree, and the focus was manually adjusted. A schematic showing the camera trajectories around the plant is shown in Figure 1.

The camera was not calibrated beforehand. It was set to program mode, which causes the camera to automatically change its shutter speed and aperture to achieve the optimal exposure. The typical parameters were as follows: F10 aperture, 1/500 s shutter speed, and an ISO value of 640. The camera was manually focused before taking the photographs. The image size was 6,016 × 4,016 pixels, and a total of 37 pictures were taken along the camera trajectory. The photography took less than 5 min and produced a total of 389 MB of image data.

Pix4DMapper (Pix4D SA, Lausanne, Switzerland) and PhotoScan (AgiSoft LLC, St. Petersburg, Russia) were used to process photographs for computer vision and photogrammetry. These are two commercial photogrammetry programs frequently used in many research applications. The image processing steps are similar in the two programs. The first step uses a structure from motion (SfM) technique to reconstruct both camera positions and 3D points. More specifically, unique features were extracted from the images and then matched with features extracted from other images. A self‐calibrating bundle block adjustment is then applied to calibrate the cameras and derive a sparse set of feature coordinates (Schönberger & Frahm, 2016). The next step is point cloud densification, where MVS (multiview stereopsis; Furukawa & Ponce, 2007) or a similar technique is used to generate a high‐density point cloud, which is color‐coded using the original image. After these two steps, a point cloud with XYZ coordinates and RGB values is generated. The whole process is easy and straightforward.

**Figure 2:**
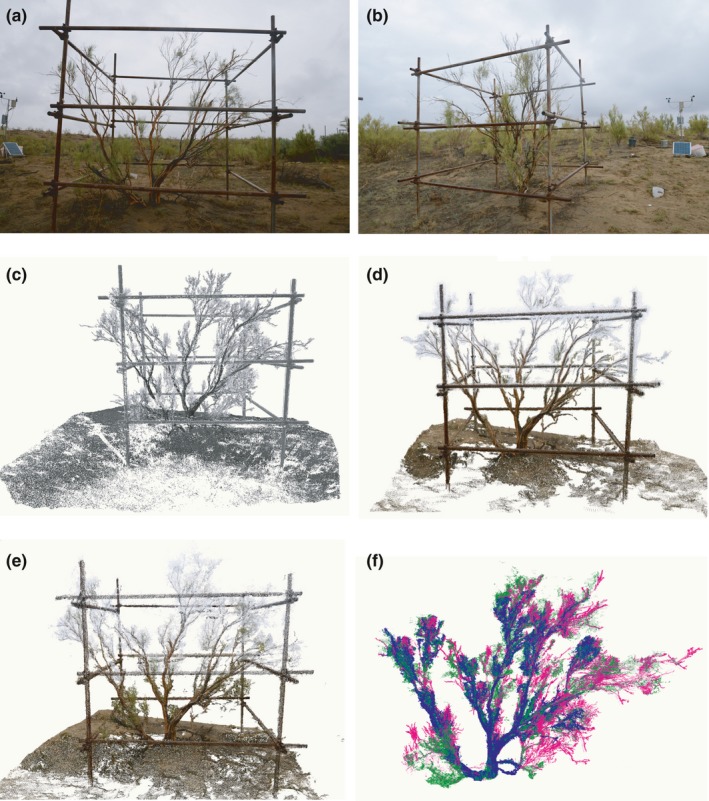
Plant photographs and the point clouds generated. (a, b) Two photographs of a plant taken from different viewing angles that were used for 3D reconstruction. (c) Point cloud data of tree 1 obtained from TLS with false color based on the reflected intensity; only the tree and its proximity are shown. (d) True‐color point cloud data derived from images processed by Pix4D (p4d). (e) True‐color point cloud data derived from images processed by PhotoScan (agi). (f) All point clouds combined (p4d points are colored blue, TLS are fuchsia, and agi are green)

We used ground control points (GCP) to transform the image's 3D data to the TLS project coordinate system. This facilitated the comparison between the image‐derived and TLS point clouds by giving both sets the same coordinate system. Five points were used as control points; four of them were at the center of the bolts that fasten the iron pipes at the four corners of the cage, and the last one was located at the upper left corner of the solar panel near the ground (Figure 2). The *XYZ* coordinates of the five points were determined manually from the merged TLS point cloud data. Based on the point density and TLS range accuracy, the accuracy of the five control points was determined to be approximately 2 cm.

Image processing time and the number of points generated are summarized in Table 1. It is evident from the table that from the 37 photographs, more than 2 million points were reconstructed by Pix4D in slightly more than 15 min. The processing time for PhotoScan was longer (more than 80 min); however, the number of points generated is also higher. It should be noted that these numbers could change based on different software versions and/or setup parameters.

**Table 1:** Image processing metrics

Processing software	Processing time (s)	Number of points	GCP error (mm)	Note
Pix4Dmapper (V 1.4)	939	2,298,189	4	Default setting with medium‐quality resolution
PhotoScan (V1.1)	4,882.87	3,695,577	7	Default setting with medium‐quality resolution
PhotoScan (V1.1)	33,863.3	11,363,407	7	Setting with high‐quality resolution

Irrelevant data points such as those corresponding to the ground and cage were manually removed from both TLS and image‐derived point cloud data, and only the points corresponding to the tree were kept (hereafter referred to as TLS, p4d, and agi, respectively). Only data with similar densities were compared (i.e., data generated with the default medium‐quality settings). Figure 2 shows the plant point cloud reconstructed from photographs using the two software packages.

## 3 RESULTS

### 3.1 Data completeness

From Figure 2, we can see that the plant and its surroundings are realistically reconstructed in the point cloud data generated from the images. With the RGB values for each point, fine and vivid details of the scene are clearly evident, such as the brown and dark trunk of the plant.

The major components of the plant (its branches and leaves) are visible in the image‐derived point cloud, but some of the finer branches and leaves are missing, particularly in the lower and the furthest parts of the tree, making it difficult to compare those regions in the image‐derived point cloud with the reference TLS data. Additionally, there are some false data points indicated in the figure by the presence of blue and green points where there are no corresponding TLS points. Noise is also noticeable in some regions; for example, at the higher part of the plant, there is a thick patch of white points. These white points can be reduced in the image preprocessing step by applying a mask to the background. A mask delineates the area of the image that will be used for 3D modeling, and the background is therefore excluded. Manual masking is a time‐consuming process and it was not implemented in this study.

We looked at the point spatial distribution pattern in different scene configurations. After deleting the apparent noise and unrelated objects from the scene, we obtained a point cloud of tree 1 along with the cage and the ground. Next, the ground and the steel pipes were manually removed. The spatial point density also changed after this process as shown in Table 2; it shows that the ground and cage bars were represented by approximately 1 million points.

**Table 2:** Point information before and after isolation of plant points

Data	Before isolation of plant points			After isolation of plant points		
	Bounding box size (m^3^)	Point number	Density (point/m^3^)	Bounding box size (m^3^)	Point number	Density (point/m^3^)
TLS	5.691 × 4.938 × 3.777	1,627,839	15,339	4.296 × 3.210 × 3.397	627,161	13,388
p4d	5.374 × 4.459 × 3.748	1,593,217	17,739	4.303 × 3.236 × 3.381	459,482	9,760
agi	5.015 × 4.268 × 4.041	1,442,423	16,677	4.179 × 3.263 × 3.718	454,270	8,960

It can be inferred from the table that the points in the image are not evenly distributed spatially; they are concentrated in the ground and the pipes. After extracting the points corresponding to the plant, the density drops to nearly half of its original value (relative difference of approximately 45%). In the TLS data, the distribution is more even, as the point density before and after removing the ground and the pipes changes very little (a relative difference of approximately 13%).

### 3.2 Accuracy assessment

**Figure 3:**
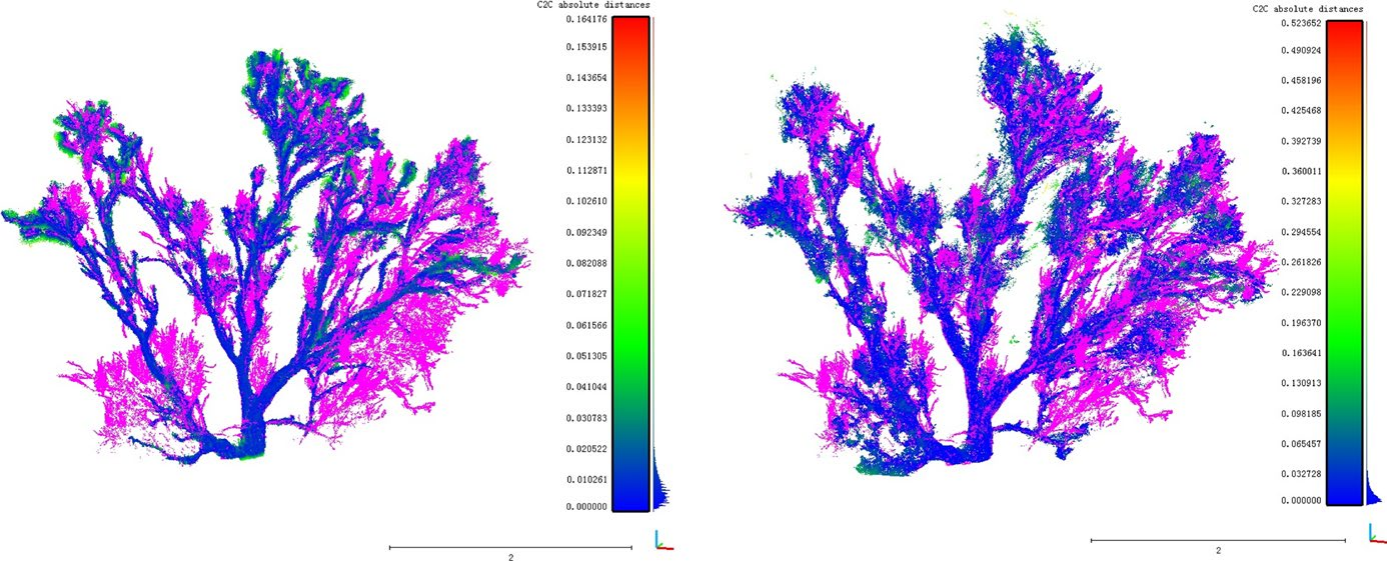
Point cloud comparison. The left image is pix4d, and the right is agi. The color bars show the distances of the compared points to the corresponding TLS points. The histogram next to each color bar shows the frequency distribution of those distances. Points in fuchsia are parts of the vegetation that did not get reconstructed from the images (TLS points which are missing from the image‐derived point clouds)

To quantify the spatial distribution of the points, we computed the cloud‐to‐cloud distance in CloudCompare using TLS data as a reference. CloudCompare is an open source 3D point cloud and mesh processing program (CloudCompare, 2017). The distances were computed relative to the reference points. The user manual suggests that the reference be denser and/or cover a larger area. Point clouds derived from the images were compared to the TLS point cloud. The program computed the distances from each cloud's points relative to the reference cloud. The results of the cloud comparisons are shown in Figure 3. When compared to the TLS data, most of the p4d points have a clear corresponding reference point within 0.01 m. The p4d data were clean, with little noise, as indicated by its 0.009 m standard deviation for cloud‐to‐cloud distance, as well as by its maximum distance of 0.16 m. The average cloud‐to‐cloud distance for agi was approximately 0.02 m; the points were more diffuse with wider ranging distances, and they had a larger standard deviation of 0.025 m. The overall quality of the two sets of image‐derived points is reasonable, considering that the accuracy of the GCP was 2 cm.

**Figure 4:**
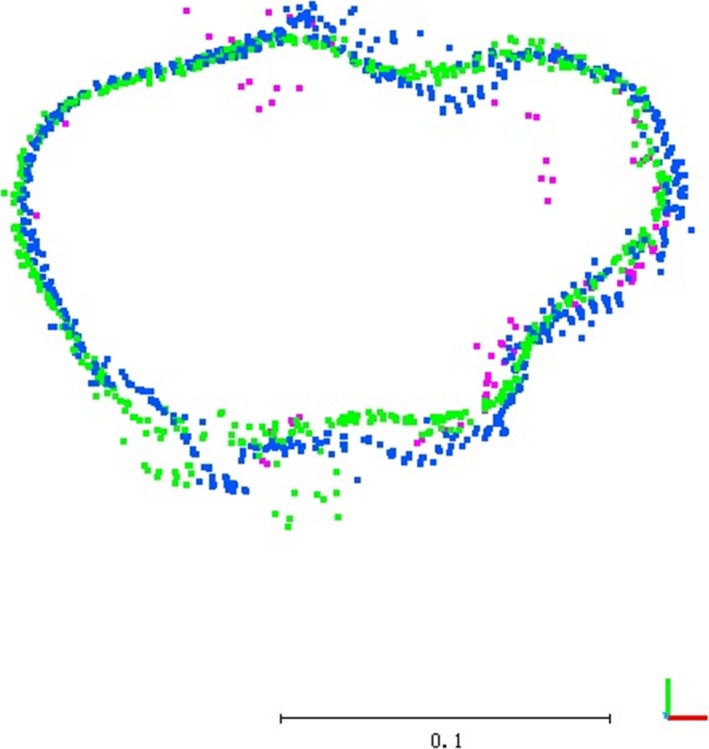
Top view of the 2‐cm slices of the point clouds corresponding to the main trunk at 0.17 m above the ground. p4d points are blue, TLS are fuchsia, and agi are green

A closer look at the point clouds revealed more detailed information about their similarities and differences. Horizontal slices (parallel to the *XY* plane) of 2 cm thickness were cut through the point cloud at different heights. Figure 4 shows the surface points of the main stem at 5% of the tree height (approximately 17 cm above the ground). Due to its irregular shape, it is not suitable to fit a circle or ellipse to these points; however, these points do show a high degree of spatial agreement visually, which is supported by the minor differences in the sizes of their bounding boxes (Table 3).

**Table 3:** Parameters of the 2‐cm slices from the point clouds corresponding to the main trunk at 0.17 m above the ground

	Point #	Bounding box size (*X* × *Y*, in m)
TLS	103	0.196 × 0.139
p4d	680	0.203 × 0.148
agi	576	0.203 × 0.153

### 3.3 Parameter extraction and 3D modeling of the point clouds

We also compared the plant parameters that could be conveniently extracted and the tree models that could be reconstructed from the different point clouds. Tree heights and crown widths were measured manually from the noise‐removed point cloud. Tree height was defined as the maximum difference in the vertical (*Z*) direction, and crown width was defined as the maximum difference in the horizontal (*XY*) plane. The parameters derived from TLS point clouds were used as reference values. Due to its protected status, we did not enter the cage to measure the plant height and crown width directly. As the TLS data were acquired from multiple positions with fine resolution, it can capture surface information of the whole plant and the plant height and crown width derived from these TLS data can be regarded as accurate. Table 4 shows that the differences between the parameters extracted from the TLS and image‐derived point clouds are very small. Therefore, good‐quality information can be obtained from the image‐derived point cloud just as it can be obtained from the TLS point cloud.

**Table 4:** Plant parameters extracted from the different point clouds

	Tree height (m)	Crown width (m)	Height abs. difference	Height relative difference	Width abs. difference	Width relative difference
TLS	3.41	4.30	NA	NA	NA	NA
p4d	3.32	4.40	−0.09	−2.6%	0.10	2.3%
agi	3.40	4.35	−0.01	0.3%	0.05	1.2%

**Figure 5:**
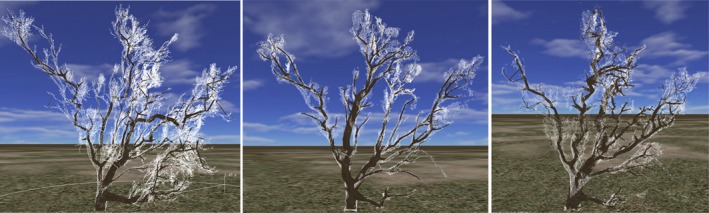
3D tree models (in black) superimposed on points (in white) created from (from left to right) TLS, p4d, and agi point clouds

To facilitate 3D modeling, an octree‐based resampling was performed on the point clouds to remove noise and make data more compact. The voxel size was 1 cm, and all points falling into an octree node are represented by the voxel center. This is followed by tree modeling (Huang, Tang, & Chen, 2015; Tang, Zhang, Huang, & Chen, 2017). The results in Figure 5 demonstrate that although the quality of the point clouds varies, 3D models can be reconstructed from both TLS and image‐derived points.

## 4 DISCUSSION

Scans by a terrestrial laser scanner from different positions paint a more complete, accurate, and detailed picture of the plant and its environment. However, this method can be expensive and time‐consuming. It took approximately 1 hr to set up and scan the plant using TLS, while the time for taking digital photographs was less than 5 min. Data processing took similar amounts of time (approximately 30 min for TLS vs. 15 min for Pix4D and 80 min for PhotoScan). Manually carrying the heavy instrument (it should be noted that there are smaller and lighter scanners available) over a long distance to a hard‐to‐reach survey site would be burdensome. It also takes some training and experience to learn how to set up scan positions and operate scan controls. In contrast, consumer‐grade handheld cameras are widely available, cheap, lightweight, and easy to use; the image processing program is also very easy to use.

To apply a computer vision (SfM) algorithm, images need to be taken with enough overlap and from different viewing angles. Following this guideline, we were successfully able to reconstruct dense surface points from the saxaul plant from 37 photographs taken with a consumer‐grade camera. Although it is easy and safe to take as many photographs as one wishes to guarantee a successful 3D reconstruction, having to process so many photographs may not always be desirable because of the additional time and computing resources required. Taking just enough photographs for 3D reconstruction remains an art as the composition of each scene is different and the number of features varies. It seems that a minimum of 60% overlap between the images is highly recommended for 3D reconstruction. Some researcher (Surový, Yoshimoto, & Panagiotidis, 2016) suggested that for a point to be correctly reconstructed, it should be captured by at least five cameras (or in five photographs).

We also reconstructed another single saxaul plant growing nearby for this experiment (results not shown in this manuscript). Unlike the plants being protected for long‐term ecological observation, it was not enclosed in a cage. Surface points of its stem and main branches were reconstructed from 20 photographs. We suspected that the regularly shaped steel pipe cage actually helped 3D reconstruction by providing more feature cues; however, even without this additional aid, saxaul plants were still effectively modeled. It will be interesting to determine whether this image‐based reconstruction also works for other desert plants such as *Tamarix ramosissima*, whose trunk is mostly covered by dense leaves during certain times of the year.

We processed these photographs with two leading commercial photogrammetry software packages using the default settings. The point clouds obtained from image processing had medium quality and density. It is possible to achieve points with better quality and density; however, the results thus far prove that these medium‐quality point clouds are comparable to the data points acquired from TLS in terms of data quantity and accuracy. The price of the software is a few thousand dollars (Table 5), which represents additional and rather significant cost. However, if compared to the around 50K USD price tag of a lightweight and less expensive TLS (as far as we know), this is still cheaper and more affordable.

**Table 5:** Cost comparison of TLS and photogrammetry software

	Terrestrial laser scanner		Photogrammetry software	
	Riegl VZ‐400	Faro Focus	Pix4Dmapper	PhotoScan
Price range (USD)	150,000	50,000+	4,500	3,499

VZ‐400 is based on the price we paid when it was purchased in 2011; software prices are for stand‐alone license, Professional Edition.

In our study, as well as in other similar studies (Fritz, Kattenborn, & Koch, 2013; Mikita et al., 2016; Miller et al., 2015) on image‐based plant reconstruction, dense leafy parts are often missing, while the main trunk and major branches are better preserved in the resulting point data. Existing computer vision algorithms have difficulty finding feature points and matching them to leaves because the leaves resemble one another, tend to occlude each other, and move easily in the wind. Currently, this method is more suitable for plants with striking, prominent branching structures or for plants that have shed their leaves. Future research and development should focus on algorithms that can detect and match feature points from both branches and leaves.

Point data derived from image matching do not have a scale, so we need to use ground control points or other distance measurements to give the point cloud a coordinate system or scale. As mentioned in the previous section, five GCPs whose coordinates are derived from the TLS scans were used in our study. However, in many circumstances (e.g., simply for distance or volume measurement purposes), this GCP procedure is not required. It would be easier to make a few distance measurements of some of the objects in the scene while one is out in the field taking photographs and later assign dimensions to these objects in the reconstructed point cloud.

In this study, we sought to recover the 3D structure of a single plant using overlapping images taken with a camera from the ground. This technique can easily be extended to wider areas covering many plants (Mikita et al., 2016). For larger‐scale applications, an unmanned aerial vehicle (UAV) could be utilized as a platform to carry a camera over an area (Cunliffe, Brazier, & Anderson, 2016; Mikita et al., 2016). Detailed spatial information on plants (location, species, size, and height) can be quickly and effectively obtained from image processing techniques.

Image‐derived points are unevenly distributed spatially; the density of a point is related to the plant structure and image frame configuration among other things. Different software and parameter settings also generate somewhat different results. More studies are needed to fully understand the advantages and disadvantages of these software programs. In addition to the *XYZ* coordinates, there are also RGB color values associated with each point. This natural color information not only can provide better visualization effects, but can also aid in point cloud processing (e.g., segmentation or classification of different objects and parts).

In addition to the commercial software, there are some open source computer vision programs such as Bundler (Snavely, 2010; Snavely, Seitz, & Szeliski, 2006), VisualSFM (Wu, 2011), and, more recently, Colmap (Schönberger, 2016). Knapitsch et al. (2017) reported that Colmap performed even slightly better than Pix4D in their benchmark testing. With these free software options and the wide availability of digital cameras (including smartphone cameras), the whole process of plant digitization via computer vision and photogrammetric software can be made easy and more affordable.

## 5 CONCLUSIONS

The purpose of this study was to determine whether detailed and accurate 3D desert plant surface data could be obtained from overlapping images taken with an off‐the‐shelf consumer‐grade handheld camera. By comparing the point clouds derived from different sources (terrestrial laser scanner and photogrammetric software output), as well as plant structural information extracted from the point clouds, we found that the image‐derived points were dense and accurate enough to represent the detailed major branching structure of the plant. However, there were missing points in some of the finer branches and leaves, and noise was present in areas with significant background interference. Compared to the reference terrestrial laser scanning data, the average accuracy of image‐derived point data was within 2 cm using point‐to‐point comparisons. The relative differences of the extracted plant heights and crown widths were below 5%. Thus, we have definite answers to the questions we posed.

Computer vision and photogrammetry software offer an easy, low‐cost, and accurate approach for 3D plant reconstruction. Terrestrial laser scanning produces accurate and complete data, but this technique is limited by its heavy and relatively expensive hardware and its more technically demanding operation. Both TLS and computer vision technology are developing fast, as lighter and more affordable LIDAR sensors are emerging. By no means will one technique totally replace the other; we just have more tools in our toolbox to choose from. We anticipate computer vision techniques will complement TLS and find more applications in ecology and environmental monitoring and research.

We expect that future research and development will focus on the automation of some parts of the processing, such as masking or scaling, plant structural parameter extraction, and the integration of the whole process into a single software package. Following in this direction, a low‐cost photogrammetric analysis toolbox could be very helpful for assessing plant growth and monitoring the environment.

## CONFLICT OF INTEREST

The authors claim no conflict of interest.

## AUTHOR CONTRIBUTION

CC and HH designed the experiment; HH and HZ acquired the data and performed the analysis; HH wrote the manuscript; CC and TL revised the manuscript.
